# hUMSC vs. hUMSC–Exosome: Which One Is Better for Epilepsy?

**DOI:** 10.3390/ph15101247

**Published:** 2022-10-10

**Authors:** Sri Hastuti, Rinaldi Idroes, Imran Imran, Yetty Ramli, Abdul Hawil Abas, Trina Ekawati Tallei

**Affiliations:** 1 Doctoral Program, Faculty of Medicine, Universitas Syiah Kuala, Banda Aceh 23111, Indonesia; 2 Department of Neurology, General Hospital Dr. Zainoel Abidin, Banda Aceh 23111, Indonesia; 3 Department of Chemistry, Faculty of Mathematics and Natural Sciences, Universitas Syiah Kuala, Banda Aceh 23111, Indonesia; 4 Department of Pharmacy, Faculty of Mathematics and Natural Sciences, Universitas Syiah Kuala, Banda Aceh 23111, Indonesia; 5 Department of Neurology, Faculty of Medicine, Universitas Indonesia, Jakarta 10430, Indonesia; 6 Department of Biology, Faculty of Mathematics and Natural Sciences, Sam Ratulangi University, Manado 95115, Indonesia

**Keywords:** epilepsy, hUMSC, exosome, therapy, review

## Abstract

Epilepsy is a disorder characterized by abnormal brain cell activity that results in seizures. It causes progressive damage to neurons. Epilepsy treatment currently focuses mostly on symptoms, which also have risks of unwanted side effects. There is currently no effective treatment to prevent epileptogenesis and the resulting neural damage. Human Umbilical Cord Mesenchymal Stem Cell (hUMSC) and exosome therapy are examples of cellular therapies that may be used to treat degenerative diseases, such as epilepsy, or cell damage. However, there is still little research on the use of hUMSCs or hUMSC–exosomes for treating epilepsy. Hence, the purpose of this paper is to compare the potential and risk of hUMSCs and hUMSC–exosomes as therapies for epilepsy. This article provides a brief summary of hUMSCs and hUMSC–exosomes in multiple aspects, such as the isolation and purification method, the mechanism of action, immunological compatibility, tumorigenicity, the risk of transmitting disease, stability upon storage, the potential of new composition with other substances, and also ethical and political issues. We conclude that hUMSCs and hUMSC–exosomes have therapeutic potential for epilepsy, with hUMSC–exosomes being safer due to their reduced immunogenicity.

## 1. Introduction

Epilepsy is a brain disease in which nerve cells fail to communicate properly, resulting in seizures, which can further cause progressive damage to neurons [1,2]. The age range of people with epilepsy is 20–70 years. The World Health Organization stated that around 50 million people in the world suffer from epilepsy, with 90% of them found in developing countries [3]. The epilepsy patient mortality rate is 2–4 times higher than in other populations, and 5–10 times higher in children [4]. There is no effective therapy to prevent epileptogenesis. Epilepsy treatment currently focuses mostly on symptoms [5]. Epilepsy treatment can also cause unwanted side effects, although it has a considerable therapeutic effect in children with refractory epilepsy, but it does not have a sufficient effect in adults [6,7,8].

Anti-seizure medications are being used to treat epilepsy, along with surgeries to remove seizure-causing brain tissue or to implant of a tiny electrical device within the body to help control seizures, and a particular diet called the ketogenic diet [9]. One of the promising new therapies for epilepsy is stem cell therapy [10]. Underlying factors such as the loss of specific brain cells in epilepsy are possibly replaced by stem cell transplantation, which also provides endogenous factors to prevent epileptogenesis, which has proven to be more effective compared to the existing therapies [7,10,11].

There are various types of stem cells, for instance: (1) embryonic stem cells, which are derived from the inner cell mass (ICM) and are able to differentiate into three germ layers (pluripotent) [12]; and (2) adult stem cells, such as mesenchymal stem cells and hematopoietic stem cells, which are multipotent and can differentiate into cells from their respective lineages [13]. Human Umbilical Cord Mesenchymal Stem Cells (hUMSCs) are primitive multipotent cells capable of differentiation into cells of different lineages [12].

hUMSCs are derived from Wharton’s jelly umbilical cord. Ethically, this type of stem cell is more acceptable to use because it is isolated non-invasively and has low immunogenicity compared to embryonic stem cells [13]. Therefore, hUMSCs have high potential as an alternative for cell therapy. The damaged or lost neurons due to epilepsy can be replaced with hUMSC [13,14].

A study showed that following neural progenitor cell transplantation into the hippocampus of a status epilepticus mouse model, these cells differentiated into mature neurons [15]. These results suggest that there exists a compatible environment for differentiation of progenitor cells in the brain damaged in chronic epilepsy. This has become the basis of new cellular therapies for the treatment of epilepsy [16]. Another study conducted in a mouse model of epilepsy injected with hUMSCs from human showed a therapeutic benefit of the transplantation regarding the development of epilepsy, likely due to the ability of the cells to produce neuroprotective and anti-inflammatory cytokines. This ability is facilitated by an intercellular communication mechanism usingexosome as biochemical secretion delivery vessels from one cell to another.

Exosome components obtained from hUMSCs (hUMSC–exosomes) also have roles in the treatment of various diseases, including nervous system diseases such as epilepsy [17]. Exosomes are cell components that function as efficient paracellular secretory organelles and play an important role in intercellular communication [18,19,20,21]. Exosomes can be a very potential therapeutic agent to replace stem cells in reducing tissue injury and increasing tissue repair [14].

To our knowledge, there are currently no articles that specifically discuss the comparison of the use of hUMSC cell therapy with hUMSC–exosomes in the treatment of epilepsy. A general comparison between stem cell therapy and exosome therapy has been written by [22] in the treatment of stroke using bone marrow mesenchymal cells (BMSC) compared to BMSC-derived exosomes, and they understood that paracrine action, which is at the center of BMSC treatment, is mostly mediated by exosomes. According to previous preclinical, experimental animal research, and combined with their findings, exosome treatment is safer than stem cell therapy, has no moral dilemmas, has a high biodistribution in the brain after transplantation, and may be utilized as a medication carrier to target information [22].

This paper aims to compare the potential and risk of hUMSCs and hUMSC–exosomes as therapy for epilepsy. This article gives a succinct overview of human umbilical stem cells (hUMSCs) and human umbilical stem cell-derived exosomes (hUMSC–exosomes) in terms of various aspects, including isolation and purification techniques, mechanisms of action, immunological compatibility, tumorigenicity, risk of disease transmission, stability after storage, potential for new composition with other substances, and ethical and political issues.

## 2. The Rise of Seizures in Brain Areas

A persistent propensity to have spontaneous epileptic seizures is a defining feature of epilepsy, which also has several negative psychosocial, neurobiological, and cognitive effects. The operational definition states that any of the following circumstances can be taken to indicate the existence of epilepsy [23]:

1. A minimum of two unexplained seizures that happen more than 24 h apart.
2. One unprovoked seizure and a likelihood of further seizures equal to the overall recurrence risk following two unprovoked seizures of at least 60% happening during the following ten years.
3. The identification of an epileptic syndrome.

For those who had an age-dependent condition but are no longer affected by it and are seizure-free, or in other situations of epilepsy for those who have been seizure-free for the last 10 years without taking any medication for the previous five years, epilepsy is deemed resolved. In high-income countries, the prevalence of active epilepsy is 5–8 per 1000 people, while it is 10 per 1000 people in low-income nations, with even higher rates recorded in rural regions [23].

Epilepsy is caused by multiple factors. Most of the time, it is unclear why this happens. Researchers have connected some forms of epilepsy to particular genes, but for most people, genes are only one component of the etiology of epilepsy. About 1 in 3 persons with epilepsy has a family member who also has the disorder. A person’s susceptibility to seizure-inducing environmental factors may be increased by specific genes [24,25]. Epilepsy can occasionally result from brain damage, such as that brought on by a stroke [26], brain tumor [27], severe head injury [28], drug or alcohol misuse [29], brain infection [30], or lack of oxygen after birth [31].

The brain areas believed to be the initial location of epileptic seizures are the neocortex, mesial frontotemporal area, and hippocampus. The hippocampus is the area of the brain that has been most studied in studies related to epilepsy. The hippocampus structure consists of the subiculum, entorhinal cortex, dentate gyrus, and hippocampus proper (Ammon’s horn with subareas Ca1, Ca2, and Ca3), where these four areas are interconnected via large unidirectional excitatory anterograde connections [32]. However, there are also some retrograde projections such as those from the entorhinal cortex to Ammon’s horn and also from Ca3 to the dentate gyrus. Circuits originating from second-layer neurons in the entorhinal cortex and their axons synapse with the dentate gyrus via perforating pathways that synapse with granule cells, and interneurons are the dominant trisynaptic circuit.

**Figure 1:**
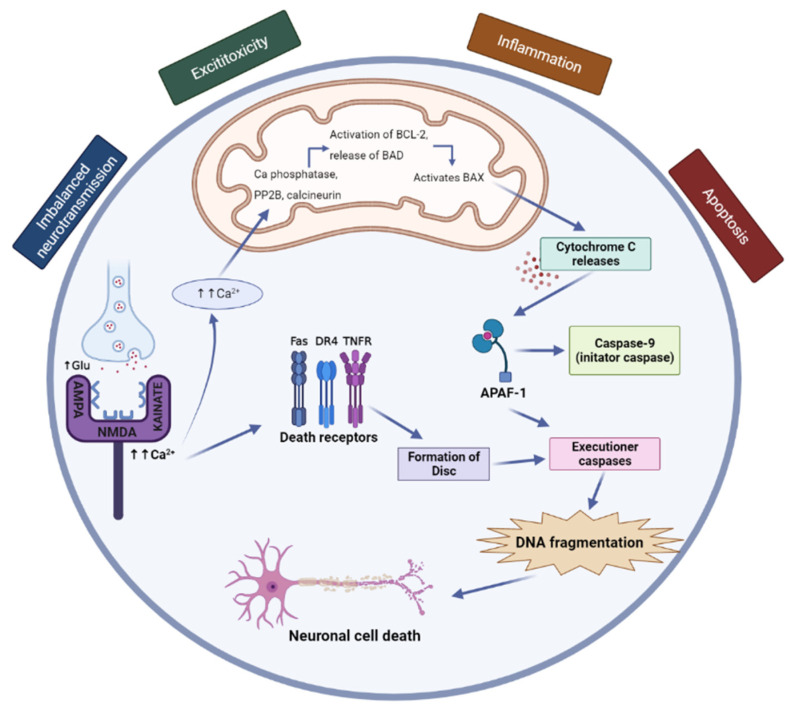
The pathophysiology of epilepsy (adapted from [35]). In the brain, glutamate serves as the main excitatory neurotransmitter, whereas GABA serves as the main inhibitory neurotransmitter. For the nervous system to operate normally, glutamatergic and GABAergic tone must be balanced. When glutamate is released in a large amount, it can cause excitotoxicity, which results in neuronal damage, cell death, and dysfunction in the remaining neurons after brain injury. These glutamates cause an increased influx of Ca^2+^ ions by overactivating NMDA receptors. By activating cytoplasmic proteases, which break down cytoskeletal and other proteins, neuronal nitric oxide synthase (nNOS), which increases nitric oxide production, and peroxynitrite, which damages DNA, the overflow of Ca^2+^ levels causes deteriorated conditions that ultimately lead to neuronal cell death. (https://link.springer.com/article/10.1007/s11910-015-0545-1 “accessed on 20 July 2022”).

Granule cells (main neurons of the dentate gyrus) project their axons to synapses in the hilus and Ammon’s horn area Ca3. Pyramidal Ca1 cells project their axons to the subicular complex and are related to the entorhinal cortex and other cortical and subcortical structures. Ca3 pyramidal cells project their axons to other Ca3 pyramidal cells, to Ammon’s horn area Ca1, and to the contralateral hippocampus [33]. The seizure leads to brain cells death in the mentioned areas, enhanced by cell apoptosis (Figure 1) [34].

Pharmacologic medications and surgery are the two current epilepsy therapy options. The main method of treatment is medication, which works by blocking sodium and other positive ion channels or by activating γ-aminobutyric acid type A (GABA_A_) receptors to decrease the total firing rate of the brain. When medication fails to control symptoms, surgical resection of the temporal lobe is an option. This procedure includes removing some of the seizure-producing brain tissue in attempt to control symptoms [36,37].

Another treatment option is vagus nerve stimulation (VNS). A tiny electrical device, similar to a pacemaker, is inserted under the skin on the chest as a part of VNS therapy. The vagus nerve, a nerve in the neck, is used by the device to transmit electrical signals to the brain. The goal is to lessen the severity and frequency of the seizures [38]. Deep brain stimulation (DBS) is yet another medical device used for treatment. In contrast to VNS, the device implanted in the chest is wired directly into the brain. By altering the electrical signals in the brain, electrical shocks transmitted through these lines can aid in the prevention of seizures. It carries some significant dangers, including the possibility of brain hemorrhage, depression, and memory issues [39].

Since the 1920s, the ketogenic diet has been utilized as a kind of therapy for uncontrollable epilepsy. The ketogenic diet has a low protein and carbohydrate intake (5%) and a high fat content (90%). Given that the type of diet should be customized for each patient and that less restrictive and more palatable diets are typically preferable options for adults and adolescents, the evidence suggests that the ketogenic diet and its variations are a good alternative for non-surgical pharmacoresistant epilepsy patients of any age [39].

Research employing CRISPRa that might control the expression of a gene for the treatment of epilepsy targets the gene Kcna1 (encoding Kv1.1) because upregulating that gene results in lower neuronal excitability, and seizures are a burst of uncontrolled electrical activity between brain cells. In a model of temporal lobe epilepsy, findings have demonstrated a decrease in spontaneous generalized tonic–clonic seizures as well as transcriptome changes related to chronic epilepsy. This work demonstrates that CRISPR is also a viable epilepsy therapy strategy [40]. Additionally, cellular therapy is one type of regenerative medicine that uses stem cells to repair a site of the body that has been diseased or damaged. Under the right circumstances, the reconstruction of the site is possible. Stem cells can be collected from several sources such as blood, fat, bone marrow, dental pulp, or from umbilcal cord like hUMSCs.

## 3. The Pros of hUMSC

Umbilical cord stem cells (UCSC) have a number of benefits over bone marrow stem cells. For starters, the collection of UCSCs is considerably easier and simpler. Blood can be drawn from the umbilical cord in as little as a few days or as late as a week [41]. Bone marrow stem cells, on the other hand, require more time to develop and collect, and their processing requires a longer period of time. This can take anywhere from a few weeks to several months. The umbilical cord blood collection procedure is painless for both the mother and the infant, requires less antigen monitoring, and reduces the danger of transmission. Bone marrow transplantation, on the other hand, necessitates that the donor be anesthetized and hospitalized after the operation [35]. Furthermore, bone marrow transplantation and stem cell preservation are costlier. In UC blood, the quantity of stem cells per unit volume is higher than in bone marrow. Graft-versus-host disease is substantially less common after transplantation with UCSC due to UCSCs’ tolerance for human leukocyte antigen (HLA) with bone marrow stem cells. Furthermore, there are advantages in terms of autologous donors due to distinct lineages that influence stem cell acceptability (such as the length of the acceptance period, and the expenses of quality monitoring and control) [42].

The first bank cord stem cell was established in 1992. It is worth noting that umbilical stem cell grafts are superior to bone marrow transplantation [43]. The quantity of umbilical cord stem cells required for a successful transplant is ten times lower than that required for both peripheral blood cells and bone marrow cells. Indeed, the hematopoietic stem cell content of 80–120 mL of blood from a single UC is similar to 1200 mL of bone marrow [43,44]. There is significant evidence that a UC contains a range of additional stem or progenitor cells, including mesenchymal stromal cells (MSCs), uncontrolled somatic stem cells (USSCs), very tiny embryonic-like stem cells (VSEL), endothelial progenitors, and epithelial stem cells [45].

## 4. hUMSC for Epilepsy

Undifferentiated cells called stem cells have the potential to multiply both as undifferentiated cells and as mature, specialized cells. Stem cell therapy is growing quickly, and several clinical trials have been started to examine the use of stem cells in the treatment of cancer, degenerative disorders, and for the regeneration of missing or injured tissue [46,47].

**Figure 2:**
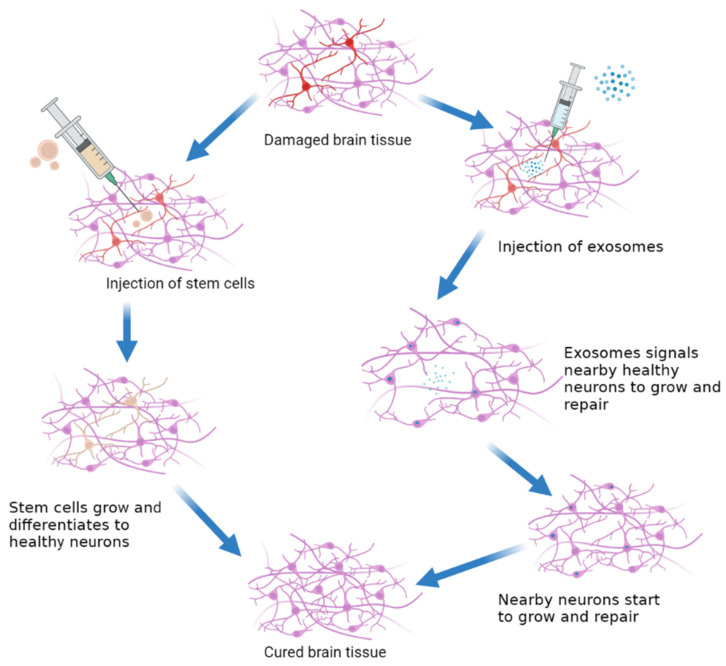
Simplified mechanism of action of stem cell therapy vs. exosomes.

In the treatment of epilepsy, stem cell transplantation works in two ways. Transplanted stem cells can replace cells lost due to trans-differentiation in the first mechanism (Figure 2). Stem cells have the potential to self-renew and specialize into numerous cell types, allowing them to replace cells or specific neurons lost during epilepsy, such as inhibitory interneurons [7]. Neuroprotection or anti-inflammatory cytokines generated by stem cells will affect the repair of autoimmune encephalomyelitis, amyotrophic lateral sclerosis, stroke, and spinal cord damage [48]. To date, it has been thought that utilizing stem cells to replace cells lost during epileptogenesis is a viable technique for treating epilepsy [49,50]. However, little is known about the epilepsy stem cell therapy’s target mechanisms. Human mesenchymal cells from the umbilical cord’s Wharton’s Jelly are collected from medical waste after delivery, posing little ethical considerations. In vitro, human umbilical cord mesenchymal stem cells (hUMSCs) can develop into neurogenic, osteogenic, chondrogenic, adipogenic, and myogenic cells [51,52,53].

Engineered hUMSCs have been found to cleave actively in the ischemic cortex, striatum, and spinal cord of mice without immune suppression in a number of prior investigations [51,54]. The ability of hUMSC to produce growth-promoting factors protects neurons from atrophy, avoids inflammation, and improves motor performance, which is most likely owing to their ability to produce growth-promoting factors [54,55]. hUMSCs isolated from Wharton’s jelly from the human umbilical cord and transplanted into the hippocampus of mice were stimulated with pilocarpine to determine if transplantation of hUMSCs in the acute phase of brain injury prevents or lowers chronic epilepsy. Several studies have found that transplanted stem cells are viable and functioning for a long period, efficiently mending hippocampus damage, lowering glial cell activation, inhibiting rearrangement of excessive electrical circuits in brain nerve cells, and suppressing brain cell inflammation [17].

In a novel in vitro model of neurons derived from individuals with Dravet syndrome (DS), the effects of human umbilical cord mesenchymal stem cell-conditioned media (hUMSC-CM) were examined. It was discovered that hUMSC-CM may efficiently reduce reactive oxygen species (ROS), influence neurogenesis and migration, and encourage neurons to achieve a highly functioning state. In light of this, they came to the conclusion that hUMSC-CM is a potential therapeutic approach for the clinical treatment of refractory epilepsy, such as DS [56].

There are various risks associated with stem cell treatment, but the two most important are the immunological response and tumor development. First, several characteristics of stem cells, such their protracted lifespan, apparent resistance to apoptosis, and capacity for lengthy replication, are similar to those of cancer cells. As a result, stem cells may be thought of as possible malignant transformation candidates. Second, the host immune system may be impacted by the delivery of stem cells. The cells sent out may directly trigger an immunological response or they may modulate the immune system [57].

## 5. hUMSC–Exosomes for Epilepsy

Because of their capacity to travel intracellularly, exosomes offer greater therapeutic potential for a wide range of disorders [13]. The advent of nanomedicine has prompted researchers to investigate the pathogenic significance of exosomal particles in a variety of disorders. In nanomedicine, tailored drug delivery systems rely on the sustained release of exosomes to exert biological activity at the desired location. Exosomes are employed to generate biological reactions as vectors or carrier molecules [14].

**Figure 3:**
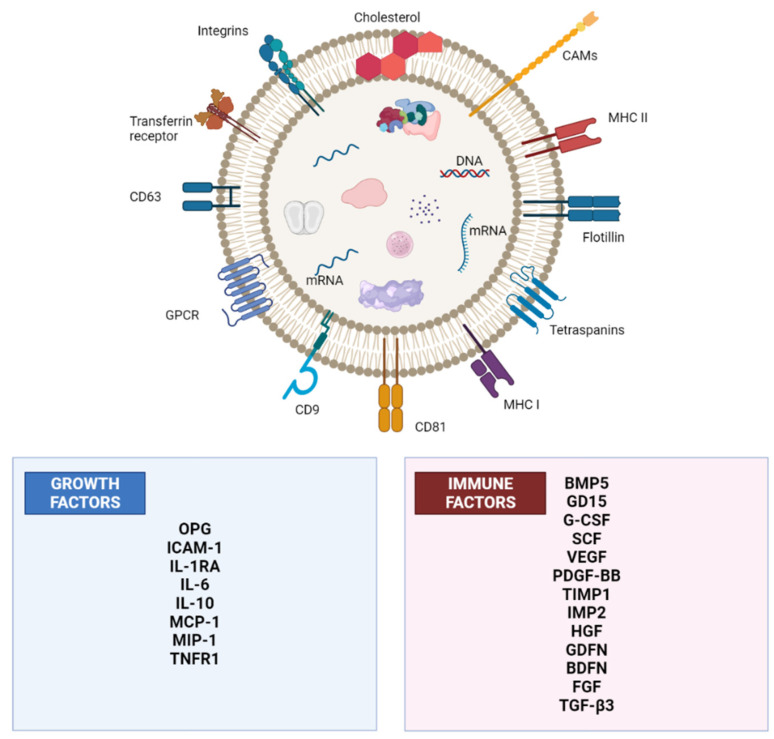
The structure of an exosome derived from a mesenchymal stem cell with its growth and immune factors (adopted from [14]).

Under certain physiological conditions, exosomes exhibit very low immunogenicity and the potential to evade the physiological blood–brain barrier [58]. With the help of a stable lipid bilayer, the cargo loaded in exosomal vesicles (EVs) is guarded against the action of immune cells and digestive enzymes. The exosomes are also identified through several proteins, including tetraspanins (CD9, CD 63, CD 81, and CD 82), heat shock protein 70 (HSP 70), ALG-2-interacting protein X (ALIX), and the tumor susceptibility gene 101(TSG101), which actually exists more in exosomes (Figure 3) [14].

**Figure 4:**
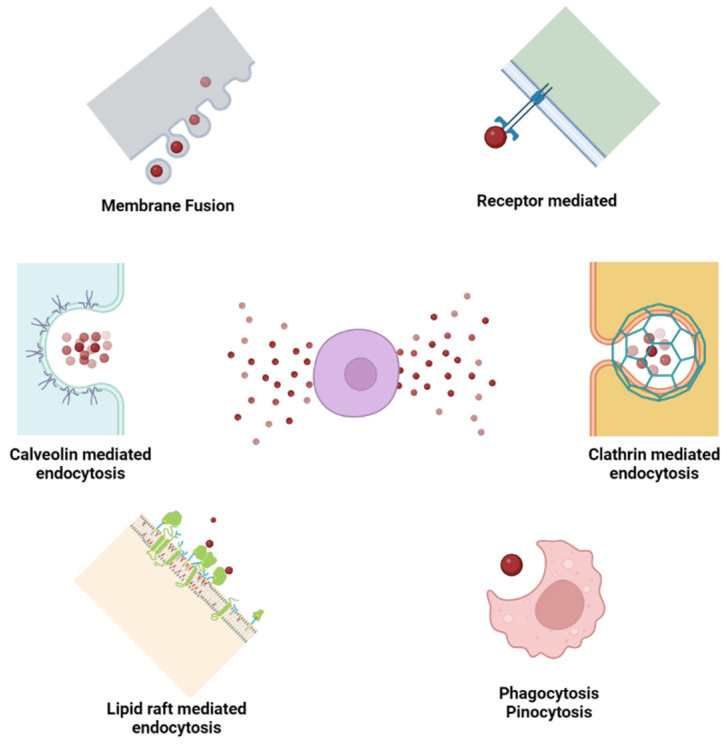
The mechanisms underlying exosomes uptake by target cells (adopted from [14]).

Endocytosis or membrane fusion are used by the modified exosomal vesicles to convey their laden cargo into the worksite (Figure 4) [14,59,60]. Vesicles can be found in a variety of cells and tissues. EVs induce tissue regeneration and homeostasis when delivered to certain sick tissues under certain settings [61]. Cell survival; trophicity; and anti-inflammatory, immunomodulatory, and therapeutic benefits are all demonstrated in EVs produced from mesenchymal stromal cells [62,63]. Neoangiogenesis and cellular proliferation are aided by them [64,65]. Exosomes show a stem cell type homing effect [14,48].

Research of hUMSC–exosomes in animal model epilepsy is still limited, even more than that of MSCs. Therefore, more research should be conducted, considering that hUMSC–exosomes have the possibility, theoretically, to work as well as MSCs (Table 1).

## 6. Comparison of hUMSCs and hUMSC–Exosomes

**Figure 5:**
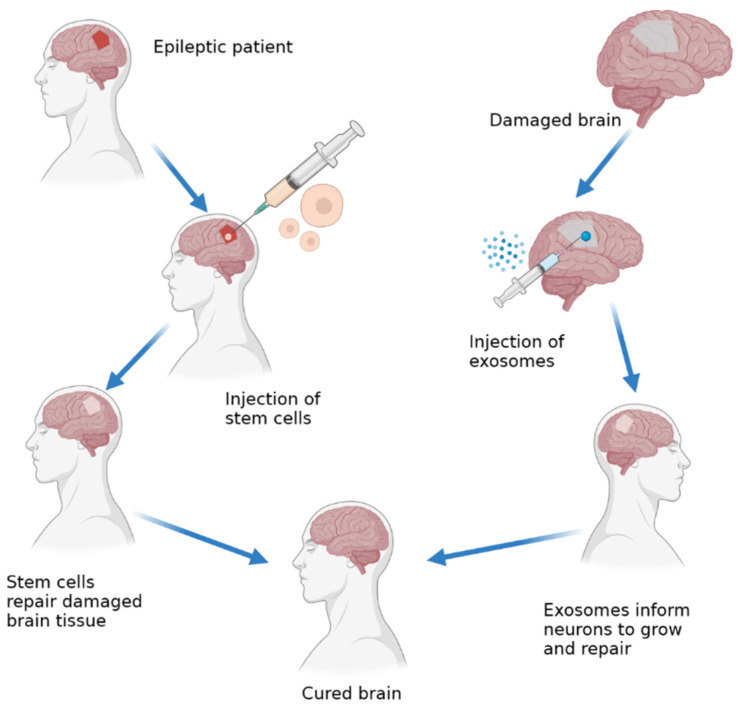
Stem cell and exosome methods of use to treat epilepsy.

Broadly speaking, the treatment of epilepsy using hUMSCs and hUMSC–exosomes does not look very different because both are methods of regenerative medicine, and the methods of use are similar (Figure 5). They are both quite different, even though they are both derived from human umbilical mesenchymal cells. Cell therapy treatment using hUMSCs uses whole cells inserted directly into the diseased area, while with hUMSC–exosomes, only the exosome is inserted. This gives the two methods some differences that can be considered in the treatment of epilepsy.

The following are some of the clinical and therapeutic benefits of exosomal loading over stem cells [64,69]:

(I) Lack of intrinsic dangers associated with any cell-based therapy, including stem cells;
(II) Lack of replication potential and risk of malignant transformation;
(III) Lack of immunogenic response to infection and cancer;
(IV) Specific actions.

The flexibility of EVs, as previously discussed, improves their intracellular signaling capabilities and their ability to travel across cellular membranes to restore micromolecular equilibrium. Exosomes, in addition to these benefits, provide neuroprotection and neuroplasticity in neurodegenerative illnesses by reciprocating across the blood–brain barrier [70]. In some countries, the use of MSCs from human still has ethical and political issues. These concerns are due to the consideration of MSCs derived from embryos. Some countries have no ethical or political issues regarding MSCs derived from the umbilical cord, because the umbilical cord is seen as biological waste and is discarded after birth [71]. As for exosomes, they has fewer ethical issues as they are not cells per se. However, the source of both MSCs and exosomes is the main consideration of ethical and political issues (Table 2).

## 7. The Route of Treatment

An intravenous infusion of MSCs reduced epileptogenesis and preserved cognitive function in a rat lithium–pilocarpine injection model of temporal lobe epilepsy by preventing neuronal cell death and lowering aberrant mossy fibers of the hippocampus [79]. In a rat pilocarpine or lithium–pilocarpine model of TLE, researchers found that intravenous infusion of bone marrow stromal cells reduced the number of seizures and saved neurons from death and neurophagia [80,81]. MSCs injected into the right hippocampus of rats with experimentally induced epilepsy reduced the amplitude and frequency of electroencephalographic spike waves and improved the dysregulated adenosine receptor expression [82]. In a rat pilocarpine TLE model, intra-hippocampal injections of human umbilical mesenchymal stem cells (hUMSCs) reduced the incidence and duration of spontaneous recurring seizures. Reduced mossy fiber sprouting, decreased neuron and interneuron loss, and inhibition of status epilepticus (SE)-induced brain inflammation are among the mechanisms driving these effects [17]. Rats transplanted with hUMSCs exhibited a longer latency time for developing spontaneous seizures, a lower seizure frequency, and a shorter seizure duration, according to the same study’s electroencephalogram recording analysis. Additionally, at the second and fourth weeks, none of the animals in the hUMSCs group had any seizure activity at all.

In a mouse pilocarpine model of epilepsy, intranasal injection of extracellular MSC-derived vesicles decreased SE-induced neuroinflammation, cognitive impairment, and abnormal neurogenesis [83,84]. MSCs’ ability to diminish epileptogenesis is partly related to their ability to protect neurons from glutamate excitotoxicity, according to in vitro studies. This protection has been linked to a reduction in the production of N-methyl-D-aspartate receptor subunits and glutamate-induced Ca^2+^ responses [85,86].

hUMSC implantation boosted the release of VEGF, induced angiogenesis [87], and had an anti-inflammatory impact by lowering the levels of inflammatory factors [88,89]. hUMSCs also had a neuroprotective impact by boosting the expression of glial cell-derived neurotrophic factor (GDNF) and BDNF and by reducing the number of hypertrophic microglia/macrophages, resulting in reduced neuronal death.

## 8. Conclusions

As cellular therapy, human umbilical cord mesenchymal stem cells (hUMSCs) are currently preferred due to their low immunogenicity and less invasive application. Despite this, hUMSC–exosomes are thought to be safer due to their decreased immunogenicity. However, the application of hUMSC–exosomes is ethically and politically more acceptable than that of hUMSCs [17,23].

Further research is required to determine whether hUMSC–exosomes or hUMSCs are the preferable epilepsy therapy option. However, before translating the basic research into clinical trials, several factors must be addressed, including regulatory guidelines, genetic testing of donors, pharmacokinetics and pharmacodynamics, and any transplantation-related concerns. Research into the long-term effects of both hUMSCs and hUMSC–exosomes to the subject are recommended. Research comparing single or more administrations of both agents and methods is also suggested to determine the optimal doses and administration methods.

## Figures and Tables

**Table 1:** Preclinical evidence of hUMSCs and hUMSC–exosomes in epilepsy and a Parkinson’s disease animal model.

Animal Model	Agents	Result	References
Pilocarpine-induced epilepsy rat model	hUMSC from Wharton’s Jelly	Intra-hippocampal transplantation of hUMSCs can suppress the spontaneous recurrent seizures in a pilocarpine TLE model. hUMSC transplantation has therapeutic benefits for the development of epilepsy.	[17]
Pilocarpine-induced epilepsy rat model	Human umbilical blood mononuclear cells	hUMCs provide prominent antiepileptic and neuroprotective effects in the experimental model of epilepsy and reinforces that early interventions can protect the brain against the establishment of epilepsy	[66]
Kainate-induced TLE rat model	GABAergic interneuron precursors from human embryonic stem cells (hESCs)	Decreased the frequency and duration of spontaneous recurrent seizures	[67]
Parkinson’s disease rats induced by 6-hydroxydopamine (6-OHDA)	hUMSC–exosomes	Exosomes were absorbed by dopaminergic neurons and microglia in the affected side, and exosome treatment inhibited microglia activation and prevented nigralstriatal dopamine neuron damage	[68]

**Table 2:** Summary of the differences between MSC and MSC–exosome therapy.

Factors	MSC–Exosomes	MSCs	References
Stress responses and immunological rejection increase the chance of necrosis.	Low risk	High risk	[72,73]
Incorporation with new compositions or methods of carrying certain substances or drugs	Exosomes can be used as carrier particles for specific components and can be combined with existing or newly created compositions or procedures	Currently, there is no method that is able to combine MSCs with other therapeutic substances	[72,73]
Tumorigenicity potential	Low risk	High risk	[72,74,75]
Isolation and purification methods	Difficulty in isolation and purification of exosomes with specific bioactive molecules	Easy to isolate and purify	
Immune problems/compatibility	Excellent immune-compatibility and non-cytotoxic	Minimal risk of immune problems	[72,74]
Ethical and political issues	Relatively free from ethical and political issues	Have ethical and political issues	[74]
Mechanism of action	Targeting efficiency through specific proteins in the exosome membranes and natural homing ability. Good delivery vehicle for both hydrophobic and hydrophilic drugs	Multilineal differentiation and highly proliferative	[72,74,76]
Stability upon freezing and thawing	Stable	Less stable	[74]
The risk of transmitting genetic and infectious diseases	No/low risk	Higher than exosomes	[74]
Previous research	Limited and insufficient research on exosome-based therapeutics	Adequate research on MSC-based therapies	[74,77]
Ability to pass the BBB (blood–brain barrier)	Exosomes pass more easily through the BBB	MSC has relative difficulty passing the BBB	[72,78]
Clearance from blood	Rapid clearance from blood after administration	Slow clearance from blood after administration	[78]

## Data Availability

Not applicable.
